# Effects of Moisture Content and Heat Treatment on the Viscoelasticity and Gelation of Polyacrylonitrile/Dimethylsulfoxide Solutions

**DOI:** 10.3390/gels10110728

**Published:** 2024-11-10

**Authors:** Jae-Yeon Yang, Yun-Su Kuk, Byoung-Suhk Kim, Min-Kang Seo

**Affiliations:** 1 Convergence Research Division, Korea Carbon Industry Promotion Agency (KCARBON), 110-11 Banryong-ro, Deokjin-gu, Jeonju-si 54853, Jeonbuk-do, Republic of Korea; yunsu@kcarbon.or.kr; 2 Department of Organic Materials & Textile Engineering, Jeonbuk National University, 567 Baekje-daero, Deokjin-gu, Jeonju-si 54896, Jeonbuk-do, Republic of Korea; kbsuhk@jbnu.ac.kr; 3 Korea Institute of Convergence Textile (KICTEX), 594 Seodong-ro, Iksan-si 54588, Jeonbuk-do, Republic of Korea

**Keywords:** polyacrylonitrile, gelation, rheological properties, moisture content, carbon fibers

## Abstract

Polyacrylonitrile (PAN) gels create significant obstacles in industrial fiber spinning by forming insoluble networks that compromise solution stability and uniformity. This study investigates the rheological properties of PAN/dimethyl sulfoxide (DMSO) solutions, examining how aging time, moisture content, and polymer concentration affect gelation behavior. Dynamic rheological analysis revealed that both physical and chemical crosslinks play crucial roles in gel formation, with gelation accelerating markedly when moisture content exceeds 3% and aging progresses. Under heat treatment at 80 °C, samples with increased moisture content demonstrated rapid transitions to solid-like states, indicating a critical moisture threshold for enhanced gelation kinetics. Additionally, reductions in polymer concentration disrupted physical crosslink density, thereby mitigating gel formation. These results underscore the importance of precisely controlling moisture and concentration parameters in PAN solutions to stabilize solution properties and minimize gel formation, thus enhancing process efficiency and quality in PAN-based carbon fiber production.

## 1. Introduction

Carbon fibers, with a carbon content of at least 90 wt.%, are valued for their high tensile strength, lightweight nature, and low thermal expansion. They are primarily derived from polyacrylonitrile (PAN), cellulose, pitch, lignin, and polyethylene (PE) precursors through stabilization and carbonization processes. Due to their desirable properties, carbon fibers are widely used in aerospace, civil engineering, military, automotive, and sports industries [1,2,3,4,5,6]. Among these precursors, PAN-based fibers are predominantly used in commercial carbon fiber production, owing to their high strength and processability.

To produce PAN-based carbon fibers, a spinning solution is prepared by dissolving an acrylonitrile-based copolymer in an organic solvent, typically followed by wet- or dry-spinning processes. These copolymers are enhanced with vinyl-based monomers containing carboxylic acid groups to improve thermal stability and flame retardancy, which is essential for high-performance fibers [7,8,9,10,11,12]. During spinning, however, gel formation in the solution can be problematic. Adding aqueous ammonia helps increase the solution’s hydrophilicity, ionizing carboxylic acid groups and enhancing solubility, but it also promotes polymer gel formation. Gel particles in the solution hinder spinning efficiency and degrade the resulting carbon fibers’ mechanical properties, posing challenges to achieving consistent quality.

To address gel formation, ethylene-based additives are often introduced, which serve as chain transfer agents, limit polymer chain length, and reduce entanglement. These additives improve solution solubility and suppress gel formation, enhancing processability. However, gelation remains an incompletely understood phenomenon, and reliable methods to control it are still lacking, limiting the stability and productivity of PAN-based carbon fibers on an industrial scale [13,14]. Gelation during processing poses significant challenges, as it affects product quality and process efficiency. Factors like temperature, pH, and polymer concentration are known to influence gelation, yet the specific role of moisture and other processing conditions on PAN solutions’ gelation behavior needs further investigation [15].

The purpose of this study is to provide an in-depth examination of the gelation mechanisms in PAN/dimethylsulfoxide (DMSO) solutions by analyzing the effects of structural changes, such as polymer chain entanglement and crosslinking, on the solutions’ viscoelastic properties. By exploring how moisture content and processing conditions contribute to gelation, this work establishes a theoretical framework to understand the interaction between environmental factors and gel formation. Specifically, dynamic rheological analysis is employed to observe gelation behavior in PAN/DMSO solutions with varying moisture contents, highlighting the structural and mechanical changes that influence gel particle formation. This study aims to fill the knowledge gap by clarifying the relationship between processing conditions and gelation, ultimately contributing to the design and optimization of stable PAN spinning processes for high-performance carbon fiber production.

## 2. Results and Discussion

### 2.1. Rheological Properties of PAN Solutions as a Function of Moisture Content

Typically, the viscoelastic behavior of a polymer solution with respect to temperature can be investigated using the loss tangent (tan δ). The time-dependent viscoelastic behavior was measured at a constant low frequency of 10 rad/s. Gelation is defined as the point where the storage modulus (G′) and loss modulus (G″) intersect, indicating a transition to solid-like behavior. This occurs when tan(δ) = 1, where the ratio between G′ and G″ becomes equal, marking the onset of gelation. Therefore, the gelation point can be expressed by the following Equation (1) [16,17,18].

(1) $$G^{\prime }=G\prime \prime$$

**Figure 1:**
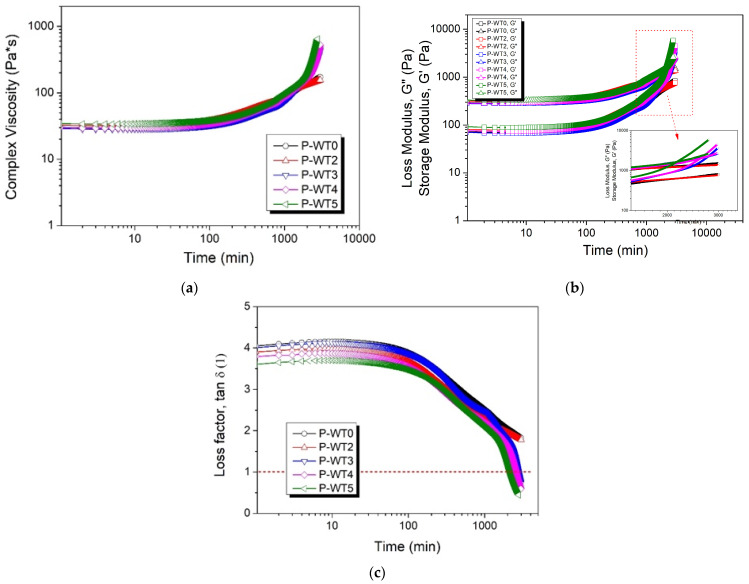
Complex viscosity (**a**), storage and loss modulus (**b**), and loss factors (**c**) of samples with different moisture contents with respect to time.

In principle, when tan (δ) is greater than 1, a liquid-like viscosity is exhibited, whereas a solid-like viscosity is dominant when tan (δ) is less than 1. The tan (δ), as well as G′ for each temperature and frequency, are shown in Figure 1. Regardless of the moisture content, all samples showed liquid-like viscous properties in the initial state and sol–gel transition regions. However, when moisture content exceeded 3%, tan (δ) became 1 or lower, indicating a gelation point [19,20]. For all samples, the complex viscosity (η*) gradually increased with increasing time. However, there were some minor differences with respect to moisture content. As shown in Figure 1 and Table 1, the PAN solution with a moisture content of 2% (P-WT2) did not form a gel [16,17,18,19,20], whereas the PAN solution containing 3% (P-WT3) started to gel, as shown by the intersection of the storage and loss moduli. Although there was no significant difference in the complex viscosity with respect to the temperature at which the moisture content was measured, rapid gelation was observed with increasing the temperature for the solutions having moisture contents of more than 3% due to the presence of water, which can accelerate the gelation. Moreover, moisture also acts as a plasticizer, which increases the mobility of polymer chains and facilitates entanglement and, eventually, gel formation [16,20,21,22].

### 2.2. Effects of Moisture Content and Heat Treatment on Rheological Properties of PAN Solutions

**Figure 2:**
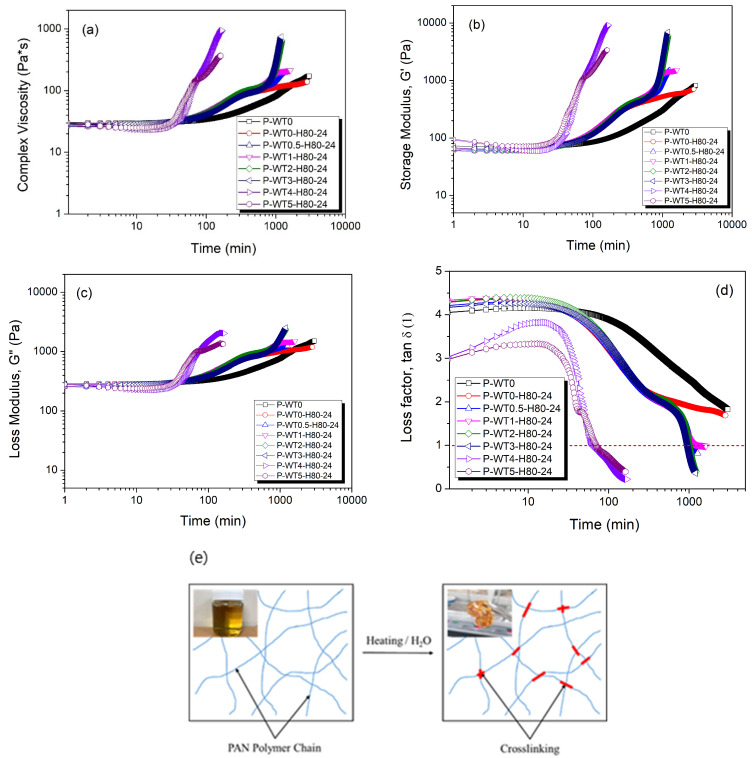
Complex viscosity (**a**), storage modulus (**b**), loss modulus (**c**), and loss factors (**d**) of the heat-treated samples with different moisture contents with respect to the time. A schematic of the gelation behavior (**e**) of PAN/DMSO solutions.

To investigate and accelerate the gelation process of PAN/DMSO solutions, samples with varying moisture contents (0% to 5%) were subjected to heat treatment at 80 °C for 24 h. The time-dependent viscoelastic behavior, presented in Figure 2a–d, shows that heat treatment significantly enhances gelation kinetics, particularly in samples with higher moisture contents. The increase in storage modulus (G′) relative to loss modulus (G″) suggests a progressive shift from a liquid-like to a solid-like viscoelastic response, as the temperature and moisture collectively promote crosslinking within the polymer. As detailed in Table 2, samples containing more than 4% moisture exhibited a pronounced phase transition to a solid-like state within approximately 1 h of heat treatment, underscoring a threshold moisture level that critically accelerates gel formation through both physical and chemical crosslinking mechanisms (Figure 2e). This observation provides insight into the moisture-driven structural dynamics within PAN/DMSO solutions, where both non-covalent (physical) and covalent (chemical) interactions distinctly contribute to the stability and rigidity of the resulting gel network.

The processes observed during heat treatment and aging can be divided as follows:

Chemical degradation: In the presence of additives, catalysts (acids and bases), and moisture, the degradation of polymer chains is catalyzed, likely due to thermally-induced scission and depolymerization reactions. This degradation modifies the molecular weight distribution of PAN, indirectly influencing the gelation kinetics by altering chain mobility and entanglement [6,9].

Physical crosslinking: Physical crosslinks arise through dipole–dipole interactions among adjacent nitrile groups in the stereoregular regions of PAN. Moisture enhances physical crosslinking by decreasing PAN’s solubility, leading to the formation of aggregates that reinforce the gel structure. As gelation progresses, crystallization and phase separation phenomena further stabilize these aggregates, resulting in a more defined network structure. This physical crosslinking is especially sensitive to moisture content, with higher levels yielding more extensive crosslink networks that increase storage modulus (G′) values relative to loss modulus (G″) [11,22,23,24].

Chemical crosslinking: Elevated temperatures, particularly in the presence of acidic or basic catalysts, promote covalent bonding between PAN chains through the nitrile group. This covalent crosslinking, or chemical gelation, is irreversible and contributes significantly to gel stability, especially in high-moisture conditions where G′ remains elevated even after extended heating. The persistence of high G′ values under these conditions highlights the role of chemical crosslinks in creating a rigid and stable gel polymer that is resistant to dilution and prolonged heat treatment [11,24,25,26,27,28].

The observed increase in G′ over G″ at higher moisture levels and temperatures reveals a pivotal balance between physical and chemical crosslinking in determining the viscoelastic properties of PAN/DMSO solutions. These findings underscore the critical importance of controlling moisture and temperature during the solution preparation and heat treatment stages. By fine-tuning these parameters, the stability and processability of PAN solutions can be optimized to minimize unwanted gel formation, enhancing both the homogeneity of the spinning solution and the mechanical properties of PAN-based carbon fiber precursors. This study’s insights contribute to a deeper understanding of PAN gelation mechanisms, with implications for improving industrial scalability and fiber quality.

### 2.3. Rheological Properties of the PAN/DMSO Solutions with Respect to Rotational Frequency

**Figure 3:**
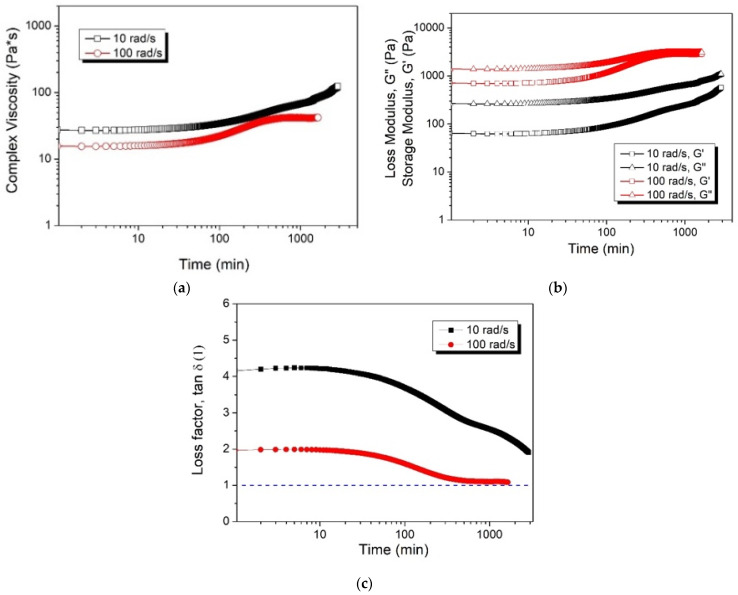
Complex viscosity (**a**), storage and loss modulus (**b**), and loss factors (**c**) of the P-WT3-H80-24 at different frequencies with respect to the time.

Figure 3 shows the rheological properties of the PAN/DMSO solution (P-WT3-H80-24) with respect to the different frequencies (10 rad/s, 100 rad/s) at 80 °C. In the low-frequency region, the PAN polymer showed liquid-like behavior in both the initial state region and sol–gel transition. As the time increased, the storage modulus in the high-frequency region gradually increased, probably due to the formation of physical crosslinks (e.g., dipole–dipole interactions between PAN chains) [29,30]. With increasing rotational frequency, the gel structure was weakened by the presence of moisture and heat treatment, resulting in low viscoelasticity. Further, the weak physical gel can also be destroyed as the rotational stress increases, as shown by the inflection point in the tan (δ) plot in the low-frequency region. This can be attributed to yielding behavior along with a slight increase in the overall viscosity [31,32].

### 2.4. Dielectric Properties of the PAN Solutions

**Figure 4:**
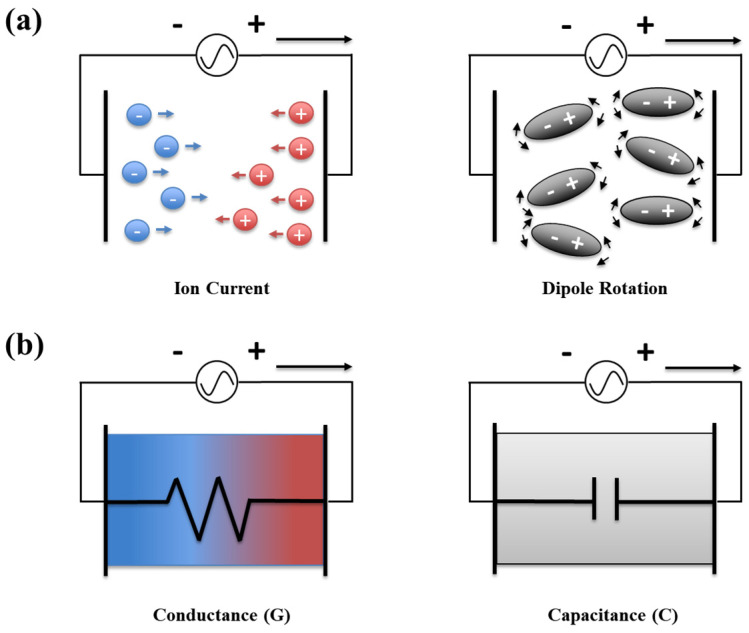
(**a**) Behavior of ions and dipoles in polymers and (**b**) ion and dipole conductions under an external electric field.

Dielectric analysis can be used for the real-time investigation of the degree of curing of dielectric materials. The conductivity (σ) and permittivity (ε) arising from ion currents and dipole rotations were measured, respectively, as shown in Figure 4a. Typically, the mobile ions in polymers are impurities or additives and separated slightly positive and negative charges produce the dipoles. Generally, when an electric field is applied to a dielectric material, the flow of ions causes a current. σ is inversely related to the resistivity (ρ), whereas the effect of mobile ions is related to the conductance (G), as shown in Figure 4b. The conductance is also frequency dependent. For instance, ion transport is easy in a material with low viscosity but becomes more difficult with increasing viscosity [33,34,35,36,37]. Specifically, when the complex viscosity of a curable polymer increases, the ionic viscosity increases correspondingly, resulting in a decrease in the ionic current. Therefore, the effects of dipoles and ions can be separated and distinguished using a dielectric analysis. Ionic viscosity is the reciprocal value of the ionic conductivity, which is calculated from the dielectric loss factor. Normally, ionic conductivity measures the extent to which mobile ions in a polymer solution can transmit charge, and it is, therefore, closely related to electrical conductivity. Unlike low molecular weight electrolytes or ionic gases, ionic conductivity in polymer solution specifically reflects the ion mobility within the polymer structure, which is based on the activity and mobility of specific ions within the polymer. Therefore, it can serve as a crucial metric for evaluating the electrochemical properties and electrical conductivity of the polymer solutions. Ionic conductivity in polymers is typically defined as the reciprocal of resistance. Therefore, a higher ionic conductivity indicates that the polymer’s mobile ions can effectively transmit the charges [33,34,35].

**Figure 5:**
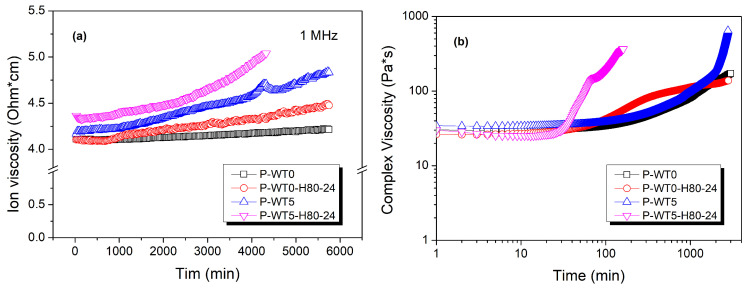
Ion viscosity (**a**) and complex viscosity (**b**) of the PAN/DMSO solutions with different moisture contents.

Complex viscosity, generally defined as resistance to deformation or its reciprocal, is influenced by factors such as temperature, pressure, concentration, molecular weight, molecular structure, and chemical state. In polymer solutions, this resistance encompasses interactions within the material, including both flow resistance and intra- and intermolecular forces that shape the viscoelastic response. For PAN/DMSO solutions, complex viscosity is impacted by structural constraints and mobility limitations arising from molecular interactions, dipole alignment, and the inherent conductivity of the polymer. In this study, dielectric measurements across a frequency range of 1 kHz to 1 MHz were employed to analyze ionic viscosity and its relationship with complex viscosity (Figure 5). The concept of “dielectric curing” describes the hardening of dielectric materials, leading to increased structural and electrical resistance, a phenomenon closely tied to gelation behavior observed in polymer solutions. In PAN/DMSO solutions with high moisture content, complex viscosity (Figure 5b) showed a gradual increase over time, similar to the trend observed in ionic viscosity (Figure 5a). This suggests that, as dielectric curing progresses, a rise in charge carrier resistance occurs, contributing to network rigidity through ionic crosslinking [33,37].

During the initial gelation stage, the physical viscosity of PAN/DMSO solutions decreases with increasing temperature, suggesting a fluid-like behavior influenced by moisture content and storage time. This decrease results from reduced intermolecular friction among polymer chains at elevated temperatures, which enhances chain mobility and delays gelation onset. However, as curing progresses, gelation accelerates, leading to a gradual increase in physical viscosity. This acceleration becomes more pronounced once a critical threshold is reached, where physical viscosity peaks, marking a transition to a more rigid, solid-like network. At this point, network formation is further evidenced by a subsequent increase in resistivity after reaching a minimum, as the developed polymer restricts ion transport, thereby raising electrical resistance. As physical viscosity rises, ionic viscosity also increases significantly, reflecting limited ion mobility within the dense polymer network. However, this increase in ionic viscosity does not directly correspond to the change in physical viscosity, indicating distinct underlying mechanisms for each. While physical viscosity primarily reflects resistance due to intermolecular interactions among polymer chains, ionic viscosity denotes the solution’s resistance to charge carrier flow under an electric field, influenced by specific ion transport dynamics [33,38,39,40]. For these reasons, it was concluded that changes in ionic viscosity in PAN/DMSO solutions with varying moisture contents are not directly proportional to changes in physical viscosity due to the distinct mechanisms underlying each property. Ionic viscosity reflects the resistance to charge carrier movement in response to an applied electric field. In PAN/DMSO systems, moisture presence enhances the ionization of PAN’s carboxylic acid groups, increasing charge carrier concentration. This ionization reduces ionic viscosity by promoting conductivity, as the increased number of charge carriers facilitates smoother ion flow. PAN naturally exhibits dipole moments due to its polar -C≡N groups, which align under an external electric field, affecting dielectric properties according to field frequency. This dipole alignment, along with ion mobility within the PAN solution, introduces frequency-dependent variations in dielectric behavior. Consequently, dielectric parameters are highly sensitive to the frequency applied; measurements in this study were conducted across a range from 1 Hz to 1 MHz at a stable temperature of 80 °C over 3 days. At the maximum frequency of 1 MHz, significant effects on dielectric properties were observed due to intensified dipole interactions, allowing for detailed insights into PAN’s dielectric responses within the polymer. In contrast, physical viscosity is determined primarily by intermolecular interactions among polymer chains, which dictate the solution’s resistance to flow. Key factors influencing physical viscosity include molecular weight, polymer concentration, chain entanglement, and the presence of additives or impurities. Elevated temperatures reduce physical viscosity by diminishing intermolecular friction, thereby enhancing polymer chain mobility. However, as gelation progresses, physical viscosity increases due to chain entanglement and network formation, leading to structural constraints within the polymer [33,34,35,36,37,38,39,40]. Thus, ionic and physical viscosities represent distinct aspects of viscoelastic behavior in PAN/DMSO solutions, each responding independently to environmental changes. Ionic viscosity is mainly associated with charge transport properties, modulated by ion concentration and dielectric behavior, whereas physical viscosity serves as a mechanical metric shaped by polymer chain interactions. The observed lack of direct proportionality between these viscosities underscores their unique contributions to gelation kinetics and structural transitions in polymer solutions.

### 2.5. UV-Visible Spectroscopy Measurements of PAN Solutions

**Figure 6:**
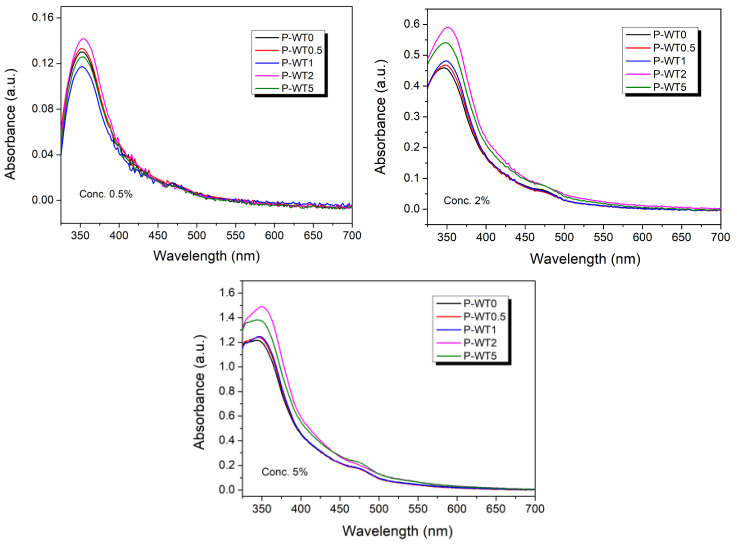
UV absorbance spectra of the heat-treated PAN/DMSO solutions with different moisture contents.

To confirm the degree of aging of the heat-treated PAN/DMSO solutions with different moisture contents, spectrometry measurements were carried out in the visible region (300–700 nm), and the results are shown in Figure 6. The height of the absorbance peak increased with increasing moisture contents, indicating a color change in the polymer solution. The structural change of the PAN polymer is likely a result of the formation of physical or chemical crosslinks in the gel due to the moisture contents and heat treatment. Moreover, it was also observed that the intensity of the absorbance peak observed in the UV-visible absorption spectra of PAN solutions increased with an increasing moisture content, which is attributed to the characteristic n→π* transition of the C≡N bond. This transition is sensitive to the presence of impurities, such as water or hydroxyl groups, affecting the electronic properties of the C≡N bond.

### 2.6. Effects of Residual Solvent on Gelation

**Figure 7:**
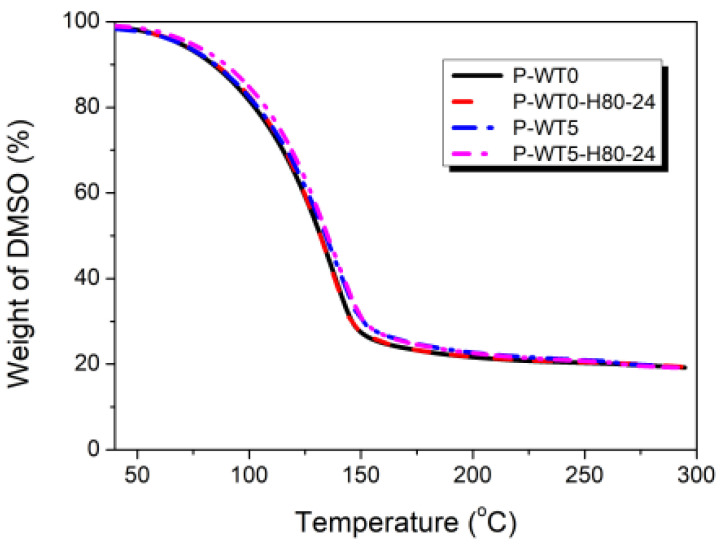
TGA thermograms of the PAN/DMSO solutions with different moisture contents and heat treatments.

TGA measurements were performed to determine the content of residual solvent in the PAN solutions. As shown in Figure 7, the solvent was rapidly volatilized up to approximately 150 °C (Figure 7). Above 150 °C, the weight of solvent was plateaued, suggesting that the solvent molecules were strongly bound to the nitrile groups of the polymer. The PAN polymer with the nitrile (-C≡N) groups possesses a polarity. These nitrile groups exhibit polarity due to the electronegativity of nitrogen, resulting in a partial positive charge on the carbon atom. Meanwhile, solvents such as DMSO (dimethyl sulfoxide) and DMF (dimethylformamide) are also polar aprotic solvents. Although they lack acidic protons, they have a polar structure that enables them to form strong hydrogen bonds with polar functional groups. Thus, the nitrile groups in PAN can engage in such interactions with these polar solvent molecules, forming hydrogen bonds. These interactions promote the solvation of PAN polymer, thereby increasing PAN’s sensitivity to solvent absorption and overall solvent interactions. This suggests that PAN solutions can undergo structural changes in PAN/DMSO solution at high temperatures, particularly when interacting with specific solvents [9,11]. Therefore, compared to the solvent molecules, the motion of the polymer chains is restricted even at high temperatures, whereas the motion of solvent molecules increases at increasing temperatures. Further, the nitrile groups in the polymer are polarized, and consequently, the repulsion between adjacent nitrile groups is enhanced. Therefore, PAN undergoes gelation and results in a change to a helical polymer structure [23,24].

### 2.7. Effects of Microparticle Structure on Gelation

**Figure 8:**
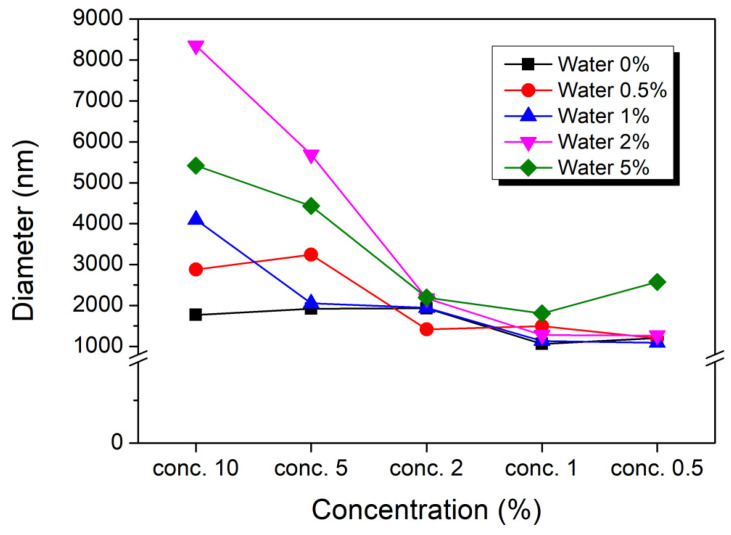
Particle size of PAN solutions with respect to the moisture content and heat treatment.

Small particles dispersed in a solution are subject to Brownian motion, where smaller particles move faster while larger particles move more slowly. In this study, dynamic light scattering (DLS) measurements were employed to monitor the size distribution of gel particles within PAN/DMSO solutions, as shown in Figure 8. To achieve consistent measurements, PAN/DMSO solutions at various concentrations (0.5% to 20%) were diluted. In solutions with the highest moisture content, microparticles exhibited the largest size, approximately 3 μm, while particle size decreased progressively as PAN concentration decreased. This result highlights a clear relationship between polymer concentration and particle size, suggesting that higher PAN concentrations facilitate gelation and aggregation, leading to the formation of larger particle structures. The formation of small, irregular gel particles can be attributed to the physical gelation process, where PAN polymer chains create an interconnected network. Differences in the orientation and interaction strength of nitrile (-C≡N) groups within PAN contribute to variations in particle size and shape, as molecular interactions are not uniformly distributed throughout the solution. Furthermore, hydrogen bonding between water molecules and PAN’s nitrile groups significantly influences intermolecular interactions, introducing additional structural irregularities within the gel network as the interactions vary spatially. It has been previously reported that aging time also affects microparticle size, with longer aging times resulting in larger particle sizes even at the same concentration [41,42,43,44,45,46]. This phenomenon can be attributed to the progressive aggregation and crosslinking of polymer chains over time as the gelation advances and the polymer network becomes denser. Additionally, dilution led to smaller particles, likely due to the partial dissolution of physical gels as the polymer concentration decreases and the solvent concentration increases. This dilution weakens the gel network, reducing particle size by diminishing gel integrity and limiting crosslinking opportunities among polymer chains [47,48,49].

### 2.8. Mass Analysis of PAN Polymer Gel

**Figure 9:**
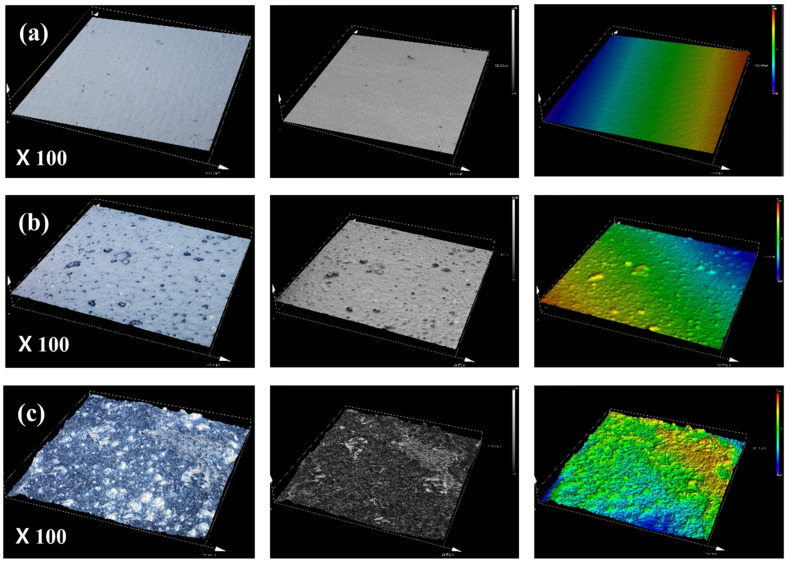
Surface roughness analysis of PAN films: (**a**) P-WT0-H80-24, (**b**) P-WT3-H80-24, and (**c**) P-WT5-H80-24.

The 3D laser confocal microscope with a short-wavelength laser was employed to capture high-resolution, non-contact, and non-destructive images of the PAN polymer surface. This method facilitated precise measurement of surface roughness and 3D morphology across scales from nanometers to millimeters, offering detailed insights into the microstructural changes within PAN films under varying moisture conditions. Measurements were conducted at ×100 magnification over 3 to 4 h following solvent volatilization at room temperature. The micrographs in Figure 9 display reflected colors, which may not represent the true color of the polymer surface. Surface roughness analysis showed that PAN films exposed to elevated moisture and heat treatment exhibited significantly higher roughness compared to untreated films. This increase in roughness is attributed to the formation of small gel particles within the polymer matrix, likely driven by intensified intermolecular interactions and crosslinking in high-moisture environments. The observed rise in surface roughness with increased moisture content suggests that moisture is crucial in promoting gel particle formation, thereby influencing the morphological complexity and network density within PAN films. Further structural analysis indicates that moisture and thermal conditions significantly impact the internal organization of the PAN polymer matrix, fostering a more interconnected and textured structure. These findings provide valuable insights into the gelation mechanisms in PAN solutions, underscoring the role of environmental factors, particularly moisture, in shaping the morphological and structural characteristics of the polymer during film formation [47,48].

## 3. Conclusions

This study provides a detailed examination of the gelation behavior of PAN/DMSO solutions under varying conditions of moisture content and heat treatment. Through dynamic rheological analysis, it was confirmed that increasing moisture content and temperature led to heightened viscosity in PAN solutions, directly supporting the hypothesis that moisture acts as a catalyst for gel formation. Specifically, samples with over 3% moisture content demonstrated a rapid transition to solid-like states when exposed to 80 °C, indicating a threshold for moisture-induced gelation.

Our results reveal that physical crosslinks, arising from electrostatic and hydrophobic interactions and hydrogen bonding, are prominent in the initial stages of gelation. However, these physical crosslinks decreased in density over time and with higher solvent content, reflecting their non-covalent and transient nature. In contrast, chemical crosslinks, due to their covalent bonds, contributed to a stable gel network that remained intact under dilution conditions, highlighting the distinct impacts of physical and chemical interactions in maintaining gel stability in PAN solutions.

The study emphasizes that controlling moisture content and polymer concentration is essential to optimizing solution stability and avoiding undesired gelation, which in turn enhances processability and the quality of carbon fiber precursors. This finding is supported by data showing that solutions with optimized moisture content maintained processability without forming disruptive gel aggregates, thus improving solution homogeneity and spinning performance.

Finally, while these results provide valuable insights, they are limited to the PAN/DMSO system. Further investigations are recommended to explore the effects of gelation mechanisms across different polymer-solvent systems to enhance the generalizability of these findings. Additionally, understanding the impacts of structural changes such as chain entanglement, branching, and crosslinking on solution processability and gel formation will be essential for advancing the production of PAN-based carbon fiber precursors.

## 4. Materials and Methods

### 4.1. Preparation of PAN Solutions

**Figure 10:**
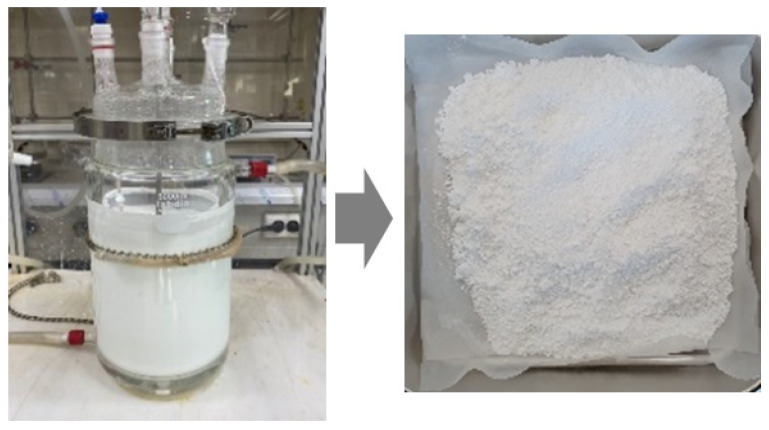
Photographs of the reactor used for the suspension polymerization of PAN polymers (**left**) and the obtained product powders (**right**).

**Figure 11:**
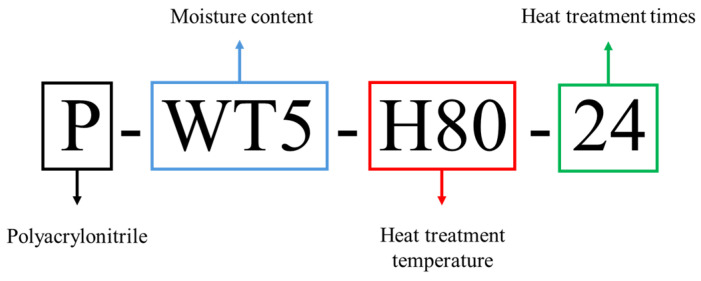
Nomenclature order of PAN solutions.

To prepare powdered PAN, the suspension polymerization method was used. The photographs of the reactor used for the suspension polymerization and the obtained product powders are shown in Figure 10. For polymerization, the concentrations of acrylonitrile (AN, Sigma–Aldrich, St. Louis, MO, USA) and itaconic acid (IA, Daejung Co., Gwangju, Republic of Korea) were fixed at 98% and 2%, respectively. The reason for using 2% IA was to introduce carboxylic acid groups into the polymer chains, which could improve the solubility of the resulting polymer in water and other polar solvents. To initiate the redox polymerization, ammonium persulfate (APS, Daejung Co., Republic of Korea) and sodium bisulfite (SBS, Daejung Co., Republic of Korea) catalysts in DMSO (Sigma–Aldrich, St. Louis, MO, USA) were used. The reaction temperature was fixed at 55 °C. After synthesis, as-prepared PAN powders were washed with distilled water and dried in a vacuum oven at 80 °C for 24 h. The obtained PAN polymer was dried again in a vacuum oven at 45 °C for 24 h before being used to remove moisture. The PAN solution (~20 wt%) in DMSO was prepared using a mixer (AR-100, THINKY INC., Laguna Hills, CA, USA). In this study, DMSO was selected as the solvent to investigate the gelation behavior of PAN solution due to its unique properties that facilitate controlled gelation studies. While N,N-dimethylformamide (DMF) is commonly used in PAN processing, DMSO’s high polarity allows for stronger dipole–dipole interactions with PAN’s nitrile groups, yielding homogeneous solutions essential for precise gelation analysis. Additionally, DMSO’s superior thermal stability under high-temperature conditions (e.g., 80 °C) minimizes premature degradation, supporting consistent assessments across varied experimental conditions. DMSO also offers a safer toxicity profile than DMF, making it suitable for laboratory and industrial applications, particularly in carbon fiber precursor research. These factors collectively establish DMSO as an effective alternative for in-depth PAN gelation studies. To determine the effect of the moisture content (WT), various water contents (0–5%) were added to the prepared PAN polymer solutions and named as P-WTx and P-WTx-H80-yy, corresponding to the moisture content (WTx) and time of heat treatment at 80 °C (H80-yy), respectively. Here, P-WT4 means that moisture content is 4 wt%. P-WT1-H80-24 means that moisture content is 1 wt%, and the sample is heat-treated at 80 °C for 24 h (Figure 11). The detailed compositions of each PAN solution are listed in Table 3.

### 4.2. Characterization

The rheological properties of the PAN polymer solutions were measured using a Rheometer (MCR 302, Anton Paar, Graz, Austria). The curing behavior of the PAN polymer was measured with time in a parallel plate configuration, and each plate had a radius of 25 mm. The gap between the plates was 1 mm, and the strain was set to ~1.0%. During measurement, the temperature was maintained at 80 °C in an oven for 2 days to accelerate gelation. The viscoelastic behavior was measured in a frequency range of 0.1–100 rad/s. Mineral oil (Sigma–Aldrich, St. Louis, MO, USA) was used to prevent the volatilization of the solvent during measurement. Two independent tests were conducted to reduce experimental errors and ensure reproducibility. The dielectric properties were analyzed using a dielectric analyzer (DEA288 ONIC, NETZSCH, Hanau, Germany) for comparison with the viscoelastic properties. The measured frequency ranged from 1 Hz to 1 MHz, and the temperature was maintained at 80 °C for 3 days using a furnace heating system. To ensure the accuracy, the PAN polymer was covered with heat-resistant tape to prevent solvent volatilization. The degree of aging was assessed using UV-spectrometry (UV-1800, SHIMADZU Co., Kyoto, Japan). The sizes of the particles formed in the polymer solutions were analyzed using a zeta potential analyzer (ELSZ-2000, OTSUKA, Tokyo, Japan). For measurements, polymer samples were diluted to the concentrations of 2%, 5%, and 10% in DMSO, respectively. Thermogravimetric analysis (TGA 550, TA Instruments LTD, New Castle, DE, USA) was used to determine the residual solvent content in the PAN polymer, and the temperature was varied from 50 to 350 °C at a heating rate of 5 °C/min. To analyze surface roughness changes from gel formation in PAN solutions, samples with varying moisture contents in DMSO were cast into films and solidified for uniform thickness. Each sample underwent a seven-point analysis using a 3D laser confocal microscope (OLS5100, OLYMPUS, Tokyo, Japan), with 3D roughness values processed through OLYMPUS software(Version 4.2 CS-ST-V4.2) to assess texture and structural variations across moisture conditions.

## Figures and Tables

**Table 1:** Complex viscosities (η*) of the PAN/DMSO solutions with different moisture contents.

Samples	Complex Viscosity (Poise) at 80 °C				Gel Point (min)
	6 h	12 h	24 h	48 h	
P-WT0	495.2	684.0	1098.6	1679.0	3000 ↑
P-WT2	564.9	733.8	1153.6	1536.7	3000 ↑
P-WT3	457.6	613.7	1133.0	3749.6	2763
P-WT4	503.4	686.4	1179.1	4450.5	2589
P-WT5	538.4	764.8	1315.8	6444.8	2150

**Table 2:** Complex viscosities (η*) of the PAN/DMSO solutions with different moisture contents under heat treatment at 80 °C with respect to time.

Samples	Complex Viscosity (Poise) at 80 °C				Gel Point (min)
	1 h	3 h	6 h	12 h	
P-WT0	320.5	386.5	495.2	684.1	3000 ↑
P-WT0-H80-24	344.2	597.8	858.1	1037.3	3000 ↑
P-WT0.5-H80-24	342.1	602.4	926.2	1174.7	1090
P-WT1-H80-24	363.0	634.2	956.1	1291.0	1370
P-WT2-H80-24	351.5	630.9	967.5	1262.5	991
P-WT3-H80-24	349.7	589.4	889.7	1232.5	963
P-WT4-H80-24	894.3	-	-	-	70
P-WT5-H80-24	1090.9	-	-	-	65

**Table 3:** Composition of PAN solutions.

Sample			PAN Solution			Moisture Content (%)
	Heat Treatment Temperature (°C)	Time (h)	PAN (g)	DMSO (g)	Water (g)	
P-WT0	80	24	20	80.0	0	0
P-WT2				78.4	0.16	2.0
P-WT3				77.6	2.4	3.0
P-WT4				76.8	3.2	4.0
P-WT5				76.0	4.0	5.0
P-WT0-H80-24				80.0	0	0
P-WT0.5-H80-24				79.6	0.4	0.5
P-WT1-H80-24				79.2	0.8	1.0
P-WT2-H80-24				78.4	0.16	2.0
P-WT3-H80-24				77.6	2.4	3.0
P-WT4-H80-24				76.8	3.2	4.0
P-WT5-H80-24				76.0	4.0	5.0

## Data Availability

The data presented in this study are openly available in the article.
